# Supplementary material for: Improving mandibular reconstruction by using topology optimization, patient specific design and additive manufacturing?—A biomechanical comparison against miniplates on human specimen
Source: PLoS One. 2021 Jun 8;16(6):e0253002. doi: 10.1371/journal.pone.0253002 (PMC8186800; doi:10.1371/journal.pone.0253002)
Supplement: S1 Table — (DOCX) [file pone.0253002.s001.docx]

| Implant type | Sex | Age | Elastic limit in N | Failure force in N | vertical displ. at failure in mm | lateral displ. at failure in mm | Stiffness in N/mm | failure type | cortical thickness at A in mm | cortical thickness at B in mm | cortical thickness at C in mm |
| --- | --- | --- | --- | --- | --- | --- | --- | --- | --- | --- | --- |
| miniplate | f | 75 | 240 | 280 | 1.78 | 0.44 | 143.5 | bone failure | 1.3 | 0.9 | 2.3 |
| miniplate | m | 62 | 350 | 470 | 1.98 | 0.78 | 192.1 | bone failure | 1.7 | 1.4 | 1.7 |
| miniplate | m | 74 | 270 | 190 | 4.06 | 0.92 | 206.5 | plate failure | 2.7 | 2.0 | 2.3 |
| TOPOS | f | 74 | 150 | 150 | 0.87 | 0.70 | 148.5 | bone failure | 1.9 | 0.9 | 1.7 |
| TOPOS | m | 74 | 400 | 540 | 3.48 | 1.60 | 137.4 | tooth failure | 2.5 | 1.7 | 2.7 |
| TOPOS | m | 63 | 280 | 510 | 3.06 | 3.82 | 168.0 | bone failure | 1.5 | 1.1 | 1.7 |

Measurement locations A, B and C can be seen in Fig 5, m=male, f=female, displ.=displacement.
