# Supplementary material for: Improving mandibular reconstruction by using topology optimization, patient specific design and additive manufacturing?—A biomechanical comparison against miniplates on human specimen
Source: PLoS One. 2021 Jun 8;16(6):e0253002. doi: 10.1371/journal.pone.0253002 (PMC8186800; doi:10.1371/journal.pone.0253002)
Supplement: S2 Table — (DOCX) [file pone.0253002.s002.docx]

| Implant type | Sex | Age | Number of passed cycles | Failure type | Actuator displ. Right in mm | Actuator displ. Left in mm | cortical thickness at A in mm | cortical thickness at B in mm | cortical thickness at C in mm |
| --- | --- | --- | --- | --- | --- | --- | --- | --- | --- |
| miniplate | m | 74 | 104300 | plate failure | 2.1 | 2.8 | 3.3 | 2.4 | 3.0 |
| miniplate | f | 79 | 51410 | plate failure | 2.5 | 3.1 | 2.7 | 2.2 | 1.4 |
| miniplate | f | 78 | 6590 | bone failure | 2.4 | 3.2 | 1.6 | 1.1 | 1.5 |
| miniplate | m | 58 | 382680 | plate failure | 1.9 | 2.9 | 2.7 | 1.4 | 1.7 |
| miniplate | f | 55 | 3760 | bone failure | 2.3 | 3.0 | 1.7 | 1.1 | 2.1 |
| miniplate | m | 65 | 11610 | plate failure | 1.7 | 2.5 | 2.4 | 2.2 | 2.0 |
| miniplate | m | 67 | 109000 | plate failure | 2.3 | 2.9 | 2.2 | 1.9 | 1.5 |
| miniplate | f | 71 | 380 | bone failure | 2.8 | 2.6 | 1.3 | 0.8 | 1.6 |
| TOPOS | m | 57 | 500000 | - | 1.95 | 2.3 | 2.9 | 1.4 | 2.4 |
| TOPOS | f | 51 | 500000 | - | 2.5 | 3.0 | 2.7 | 1.8 | 2.3 |
| TOPOS | m | 69 | 277620 | bone failure | 2.5 | 3.1 | 2.0 | 1.1 | 1.3 |
| TOPOS | m | 61 | 500000 | - | 1.8 | 2.6 | 3.2 | 1.9 | 2.8 |
| TOPOS | f | 79 | 100 | bone failure | 2.4 | 2.8 | 1.1 | 0.9 | 1.0 |
| TOPOS | f | 79 | 500000 | - | 1.9 | 2.5 | 2.4 | 1.9 | 2.7 |
| TOPOS | f | 59 | 500000 | - | 1.8 | 2.8 | 3.0 | 2.3 | 2.2 |

Measurement locations A, B and C can be seen in Fig 5, m=male, f=female, actuator displ.=displacement of the load applicators at cycle  100
