# Supplementary material for: Improving mandibular reconstruction by using topology optimization, patient specific design and additive manufacturing?—A biomechanical comparison against miniplates on human specimen
Source: PLoS One. 2021 Jun 8;16(6):e0253002. doi: 10.1371/journal.pone.0253002 (PMC8186800; doi:10.1371/journal.pone.0253002)
Supplement: Attachment — Submitted filename: Response to Reviewers.docx [file pone.0253002.s004.docx]

Vorstand:

Prof. Dr. Markus Schwaiger

(Ärztlicher Direktor, Vorsitzender)

Dr. Elke Frank

(Kaufmännische Direktorin)

Silke Großmann

(Pflegedirektorin)

Prof. Dr. Bernhard Hemmer

(Dekan)

Bankverbindung:

Bayer. Landesbank Girozentrale

BIC: BYLADEMM

IBAN: DE82 7005 0000 0000 0202 72

USt-IdNr. DE 129 52 3996

**Klinikum rechts der Isar
Anstalt des öffentlichen Rechts**

**Klinik für Orthopädie**

**und Sportorthopädie**

Mitglied des

**Jan Lang, M.Sc.**

**jan.lang@tum.de**

Ismaninger Straße 22
81675	München
E-Mail: ortho@mri.tum.de
Tel: 089 4140-22 71
Fax: 089 4140-48 49

[www.mrio.de](http://www.mrio.de)

Klinikum rechts der Isar · MOR · 81664 München

Munich, 18.05.2021

**Response to Reviewer**

Dear Mr. Cordova,

dear Mr. Mommaerts,

dear Mr. Carrasco,

thank you very much für the second revision of our manuscript entitled “Improving mandibular reconstruction by using topology optimization, patient specific design and additive manufacturing? – A biomechanical comparison against miniplates on human specimen”. In the following I would like to respond to each point mentioned in your revision.

*1.* *Please review your reference list to ensure that it is complete and correct. If you have cited papers that have been retracted, please include the rationale for doing so in the manuscript text, or remove these references and replace them with relevant current references. Any changes to the reference list should be mentioned in the rebuttal letter that accompanies your revised manuscript. If you need to cite a retracted article, indicate the article’s retracted status in the References list and also include a citation and full reference for the retraction notice.*

The reference list has been checked. All references are accessible and not retractions are present. During the two revision cycles the following references position have been added to the reference list: 10-18, 35-37

*2. As a general comment, the revision of the final revised version (PONE-D-20-39788R1) was complex because it doesn't include changes highlighted in red or yellow color. All changes were presented at the end of the compiled PDF file document as raw tracked changes. TO MAKE THE NEXT REVISION EASY, PLEASE, BE SURE TO HIGHLIGHT FINAL MODIFICATIONS IN RED COLOR ONLY IN THE FINAL REVISED VERSION*

I am sorry to hear about these inconveniences. I did use the normal Track changes function in Word, which includes a highlighted mark-up, as stated in the requirements for submission of revisions. Somehow, in the transition to the compiled pdf there must have been a problem with these markups. Nevertheless, in my pdf-summary of the upload the mark-up was tracked and highlighted normally. I did upload now tracked changes files as pdf in order to prevent this complication. If there is still a problem with the mark-up, please explain me, how exactly to solve this.

*Comments by Reviewer 1*

*3. Reviewer 1: Line 135: is high stiffness (= high brittleness) correctly the goal? Would reduced plasticity and increased elasticity not be a better criterion? Please explain in your text.*

Brittleness as a material property often comes together with a high material stiffness.

But in this case, the optimization goal was to maintain a high stiffness for the whole implant despite significant material reduction. The Young’s moduli, which are a measure for elasticity and can be seen as the material stiffness, are very similar for titanium grade 4 and grade 5. So, the material properties are not changed and the implant still has a ductile mechanical behavior. The created geometry is optimized to allow as little deformation as possible with the given material. This is criterion is chosen to have a better distribution of internal stress in the implant and to improve fatigue properties. An optimization of the implant to higher elastic deformation would have been possible, but this always comes with a decreased stiffness for the implant. In consequence the reconstruction is not as stable and the risk of interfragmentary motion is higher, which impedes proper bone healing.

*Reviewer 1: Then please explain the importance of stress shielding, the micro strains involved related to the Utah paradigm..*

Thank you very much for pointing this out. To emphasize for the reader, that stress shielding and bone loss due to reduced micro strains is not an issue for these implants, two sections were added to the manuscript. First, the optimization process was rephrased to show that an optimization goal for high stiffness does not result in an extraordinary stiffness for the implant. Secondly it was highlighted that the mechanical similarity of the reconstruction does also mean similar mechanical stresses for the fracture area.

The following sentence was added to the Material&Methods section for clarification:

“… In other words, the optimization is a material reduction process that results in the least possible loss of stiffness for the implant.”

The topic concerning stress shielding and micromotions in the fracture is now addressed with the following paragraph in the discussion section:

“The mechanical similarity also confirms that there is no excessive stiffening caused by the TOPOS implants. If the bone fragments are positioned too rigidly for osteosynthesis, it can result in inhibition of sufficient micromotions in the fracture gap [35,36]. Stress shielding like effects can lead to impaired healing in this context due to low interfragmentary strains at the fracture site [37]. But as already described there is no indication for this found in mechanical testing. “

*4. Reviewer 1: Line 180: name of device, name of company, city, country*

Thank you very much for this hint. The test system has already been described as custom made in a previous paragraph. To avoid confusion for the reader, this has been pointed out again in the manuscript.

*Reviewer 1: Difficult to confirm the results by a re-test by a third party. Please describe the flaw.*

The following sentence was added to the manuscript for clarification:

“The biomechanical testing in this study was performed on an in-house developed testing setup. Using a custom-made solution like this comes with the disadvantage of limited reproducibility for third parties. But a commercially available solution does not exist for this testing scenario. And due to the detailed description of the testing and the publication presenting the experimental setup by Foehr et al. [26], distinct transparency, comprehensibility and adaptability of the experiments are ensured.”

We hope the revised version of our article meets all the conditions for publication in your journal.

Kind regards,

Jan J. Lang
